# Convolutional neural networks versus radiologists in characterization of small hypoattenuating hepatic nodules on CT: a critical diagnostic challenge in staging of colorectal carcinoma

**DOI:** 10.1038/s41598-020-71364-5

**Published:** 2020-09-17

**Authors:** Korosh Khalili, Raymond L. Lawlor, Marina Pourafkari, Hua Lu, Pascal Tyrrell, Tae Kyoung Kim, Hyun-Jung Jang, Sarah A. Johnson, Anne L. Martel

**Affiliations:** 1 Department of Medical Imaging, University of Toronto, Toronto, ON Canada; 2 Joint Department of Medical Imaging, Sinai Health System, Women’s College Hospital, Princess Margaret Cancer Centre, University Health Network, 610 University Ave, Toronto, ON M5G 2M9 Canada; 3 Department of Statistical Sciences, University of Toronto, Toronto, ON Canada; 4 Department of Medical Biophysics, University of Toronto, Toronto, ON Canada

**Keywords:** Liver cancer, Colon, Surgical oncology, Colorectal cancer

## Abstract

Our objective was to compare the diagnostic performance and diagnostic confidence of convolutional neural networks (CNN) to radiologists in characterizing small hypoattenuating hepatic nodules (SHHN) in colorectal carcinoma (CRC) on CT scans. Retrospective review of CRC CT scans over 6-years yielded 199 patients (550 SHHN) defined as < 1 cm in diameter. The reference standard was established through 1-year stability/MRI for benign or nodule evolution for malignant nodules. Five CNNs underwent supervised training on 150 patients (412 SHHN). The remaining 49 patients (138 SHHN) were used as testing-set to compare performance of 3 radiologists to CNN, measured through ROC AUC analysis of confidence rating assigned to each nodule by the radiologists. Multivariable modeling was used to compensate for radiologist bias from visible findings other than SHHN. In characterizing SHHN as benign or malignant, the radiologists’ mean AUC ROC (0.96) was significantly higher than CNN (0.84, *p* = 0.0004) but equivalent to CNN adjusted through multivariable modeling for presence of synchronous ≥ 1 cm liver metastases (0.95, *p* = 0.9). The diagnostic confidence of radiologists and CNN were analyzed. There were significantly lower number of nodules rated with low confidence by CNN (19.6%) and CNN with liver metastatic status (18.1%) than two (38.4%, 44.2%, *p* < 0.0001) but not a third radiologist (11.1%, *p* = 0.09). We conclude that in CRC, CNN in combination with liver metastatic status equaled expert radiologists in characterizing SHHN but with better diagnostic confidence.

## Introduction

Colorectal cancer (CRC) has the 3rd highest incidence among malignancies worldwide with over 1 million patients diagnosed annually^1^. Computed Tomography (CT) scan is the most commonly used imaging modality in patients with CRC in high and upper-middle income countries and is the only modality recommended by the National Comprehensive Cancer Network (NCCN) for the routine staging and follow-up of the disease^2^. Unfortunately, small hypoattenuating hepatic nodules (SHHN), also called “too small to characterize”, occur in 16.7–25.9% of staging CT scans in patients with CRC and between 9.2 and 14.0% of these are eventually found to be malignant^3–6^. The discovery of SHHN leads to additional work-up imaging by ultrasound or MRI, or additional follow-up and adds to delays, costs and importantly, patient anxiety. With the advancement of chemotherapy for CRC, surgical and ablative therapies are now applied to treat multiple hepatic metastases in ever-growing and multi-staged combinations^7^. The result is that in most patients, each nodule discovered merits its own evaluation since interventions are tailored to treat each and every metastasis individually.

Recently, the advancing field of artificial intelligence has found increasing application in medical imaging^8–11^. Convolutional neural networks (CNN), which are based on the architecture of the human visual system, are able to learn local features directly from the input images without relying on any expert domain knowledge^12^. CNN have been shown to outperform more traditional methods in a wide variety of medical image classification problems. In the liver, radiomics and CNN have seen limited but promising application in the categorization of hepatic nodules^13–15^. However, prior studies have been limited by use of heterogeneous patient populations and/or with assessment of nodules with varied imaging patterns, sizes, and histopathologies. We hypothesize that a focused application of CNN would improve its performance to clinically relevant levels but with better diagnostic confidence than radiologists. In CRC patients with SHHN, the relatively homogeneous pre-risk probability, limited size of SHHN (< 1 cm by definition), and binary categorization (benign or malignant) may improve the performance of CNN. The potential benefit is an easily-developed tool that may answer an immediate clinical need. We therefore undertook a study with the purpose of determining the diagnostic performance and diagnostic confidence of CNN as compared to expert radiologists in the characterization of SHHN on contrast enhanced CT in patients with CRC.

## Results

### Diagnostic performance of CNN versus radiologists

Tables 1 and 2 summarize the results and diagnostic performance of CNN and radiologists using the testing set. The radiologists’ AUC ROC ranged between 0.90–0.94 and was significantly higher than that of CNN (0.84). Sensitivity, specificity and accuracy of radiologists were calculated based on their binary categorization of benign or malignant. The diagnostic accuracy of the radiologists ranged between 84.8 and 89.1% and higher than CNN’s 78.3%.

**Table 1:** Per patient results of radiologists, CNN and CNN with liver metastatic status.

SHHN	Truth	Radiologist 1	Radiologist 2	Radiologist 3	CNN	CNN & liver metastatic status
Benign	33	32	33	21	23	37
Malignant	14	13	12	22	15	8
Both	2	4	4	6	11	4

**Table 2:** Diagnostic performance of radiologists, CNN and CNN with liver metastatic status, n = 138 nodules.

Reader	AUC ROC	Accuracy	Sensitivity (CI)	Specificity (CI)	*p* value compared to radiologist mean AUC ROC
Radiologist 1	0.90	89.1	89.7 (84.6, 94.8)	88.1 (82.7, 93.5)	0.001
Radiologist 2	0.94	88.4	85.2 (79.3, 91.1)	90.5 (85.6, 95.4)	0.4
Radiologist 3	0.93	84.8	98.1 (95.8 , 100)	76.2 (69.1, 83.3)	0.09
Radiologist mean	0.96	86.2	94.4 (90.6, 98.2)	81.0 (74.5, 87.5)	_ _ _ _
CNN	0.84	78.3	81.5 (75.0, 88.0)	76.2 (69.1, 83.3)	0.0004
CNN & liver metastatic status	0.95	90.6	81.5 (75.0, 88.0)	96.4 (93.3, 99.5)	0.9

*AUC ROC* area under the curve of receiver operating curve, *CI* confidence interval.

### Diagnostic performance of CNN with other CT findings versus radiologists

In the testing set, 12/49 patients (55 nodules) had a synchronous definitive ≥ 1 cm liver metastasis. As well, 6/49 patients (32 nodules) had a synchronous extrahepatic distant metastatic disease. In all cases there was agreement between the original CT report and subsequent review of the imaging. An accompanying CT scan of chest was missing in 19/49 (38.8%) of patients and therefore pulmonary metastases were not included in the analysis. Synchronous extrahepatic metastatic disease was not a significant predictor of malignancy (OR 0.48, 95%CI 0.09, 2.68, *p* = 0.41) in a 3 variable multivariable logistic regression model and thus was dropped. Both CNN and presence of synchronous hepatic metastases reached significance and were included in a 2 variable model. There was an increase in odds ratio of 1.70 (95%CI 1.34, 2.16, *p* < 0.001,) for SHHN being a metastasis for every 10% increase in CNN calculated probability. The presence of synchronous ≥ 1 cm hepatic metastasis imparted an odds ratio of 83.4 (95%CI 17.7, 393.1, *p* < 0.001) for SHHN being a metastasis.

The diagnostic performance of CNN with liver metastatic status was calculated using the below formula:

$$\mathrm{log}\left(\frac{{p}_{i}}{1-{p}_{i}}\right)= {\beta }_{0}+{\beta }_{1}{X}_{1}+{\beta }_{2}{X}_{2}$$

${p}_{i}$ is the probability of malignancy, i.e. P(Y = 1), where Y is the gold standard. ${X}_{1}$ is the CNN derived probability of malignancy, and ${X}_{2}$ is an indicator variable (${X}_{2}$= 1 when presence of synchronous ≥ 1 cm hepatic metastasis imparted, and 0 if not). Table 2 summarizes the results of diagnostic performance of CNN combined with hepatic metastatic status, showing significant improvement from CNN alone (AUC ROC 0.95 vs 0.84, *p* = 0.0002) and not different from mean radiologist performance (*p* = 0.9) or radiologists 1, 2, or 3 (*p* = 0.06, 0.6, 0.4 respectively).

### Analysis of agreement and diagnostic probability/confidence of CNN versus radiologists

**Figure 1:**
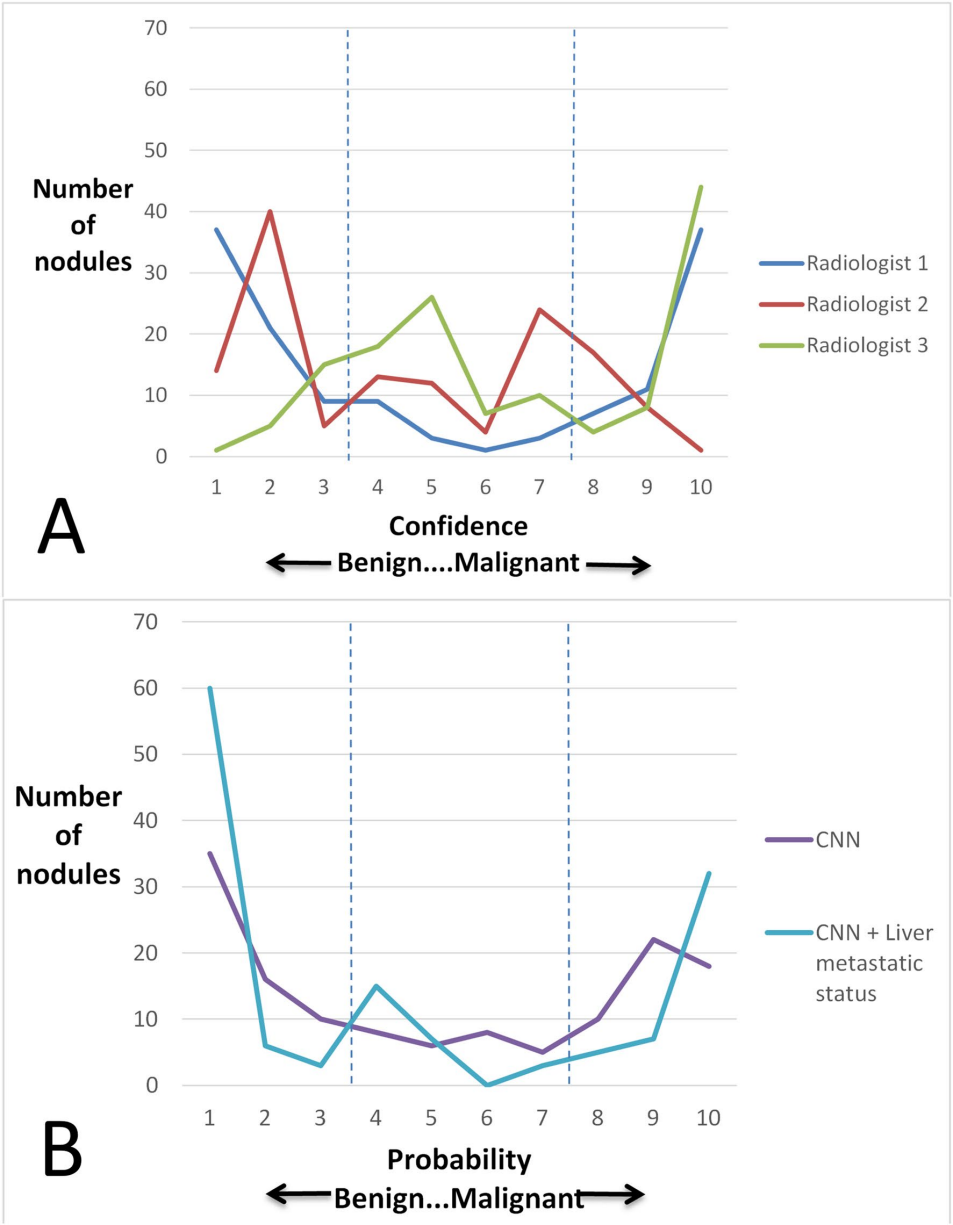
Histogram of assigned confidence/probability assigned to each nodule by radiologists (**A**) and CNN/CNN & liver metastatic status (**B**). Dashed lines outline the central low confidence zone. Radiologist 1 as well as CNN and CNN with liver metastasis status show a roughly parabolic distribution with the latter showing much steeper slopes at both extreme ends of the confidence scale.

Agreement between radiologist and CNN is summarized in Supplementary Table S2 and showing moderate to substantial agreement among the radiologists and between individual radiologists and CNN. Figure 1 depicts the distribution of confidence for the diagnosis for the three radiologists and CNN without and with liver metastatic status. The proportion of nodules that were rated in the low confidence zone (4, 5, 6, or 7 out of 10) were 16/138 (11.6%), 53/138 (38·4%), and 61/138 (44.2%) for radiologists 1–3 respectively. Figure 1B shows the distribution of probabilities calculated by CNN and converted to an ordinal scale (1–10) for means of comparison to radiologists. The proportion of nodules that were rated in the low confidence zone (4, 5, 6, or 7 out of 10) were 27/138 (19.6%), 25/138 (18.1%) for CNN and CNN with liver metastasis status respectively. There was significant difference in the proportion of nodules in the low confidence zone between CNN with liver metastasis status and radiologist 2 & 3 (*p* < 0.0001 for both) but not radiologist 1 (*p* = 0.09).

### Visualizing the CNN

**Figure 2:**
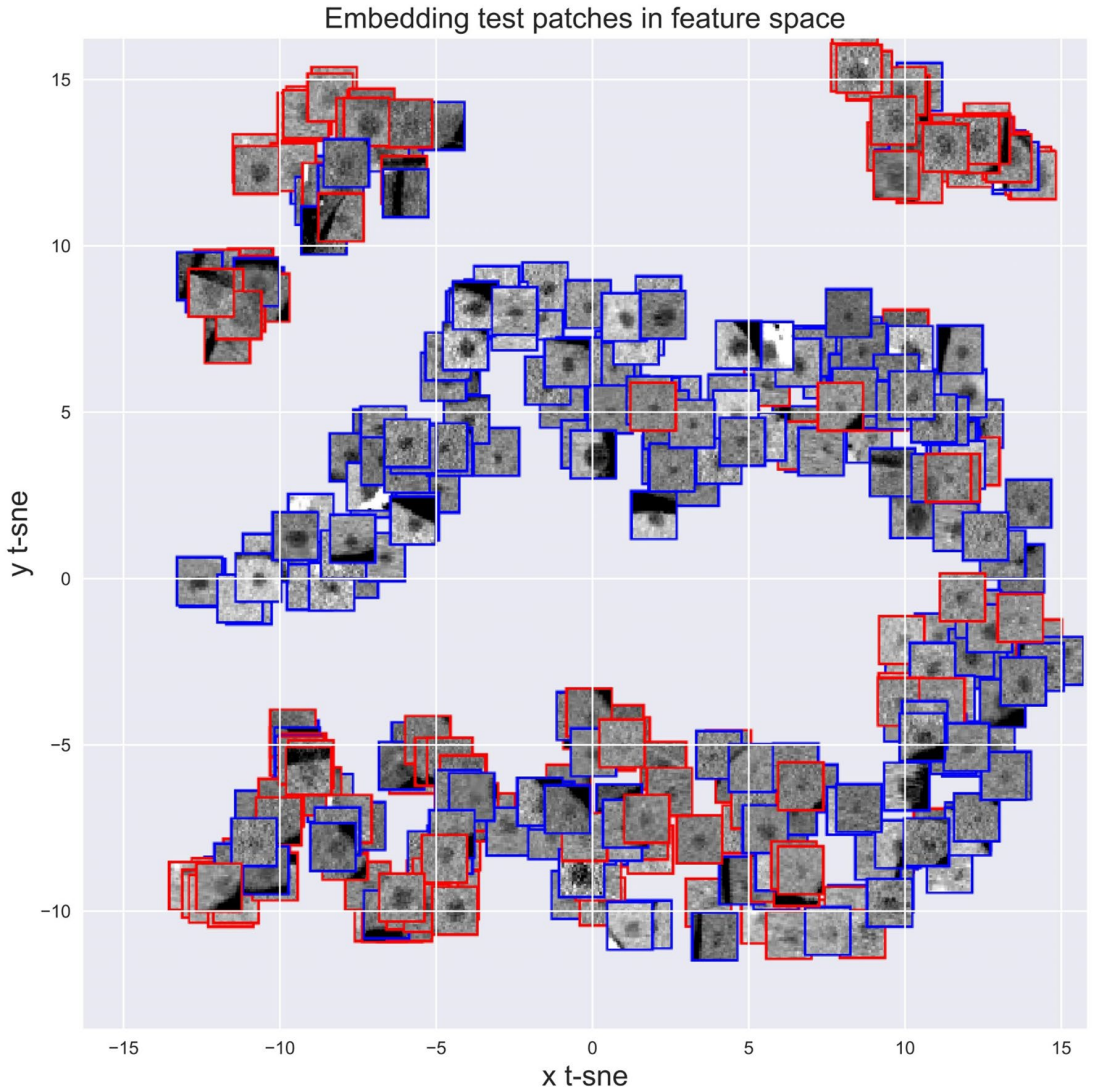
t-SNE plot of CNN nodule classification from testing dataset. The color of boxes outlining the patches indicates the actual diagnosis (blue: benign; red: malignant). Nodules rated with high probability of benignity are clustered in the left aspect of the upper arm of the reverse “C” like distribution in the center of the image. The probability of malignancy progressively increases along the lower arm of the large reverse C-shaped cluster and then within the two top clusters.

**Figure 3:**
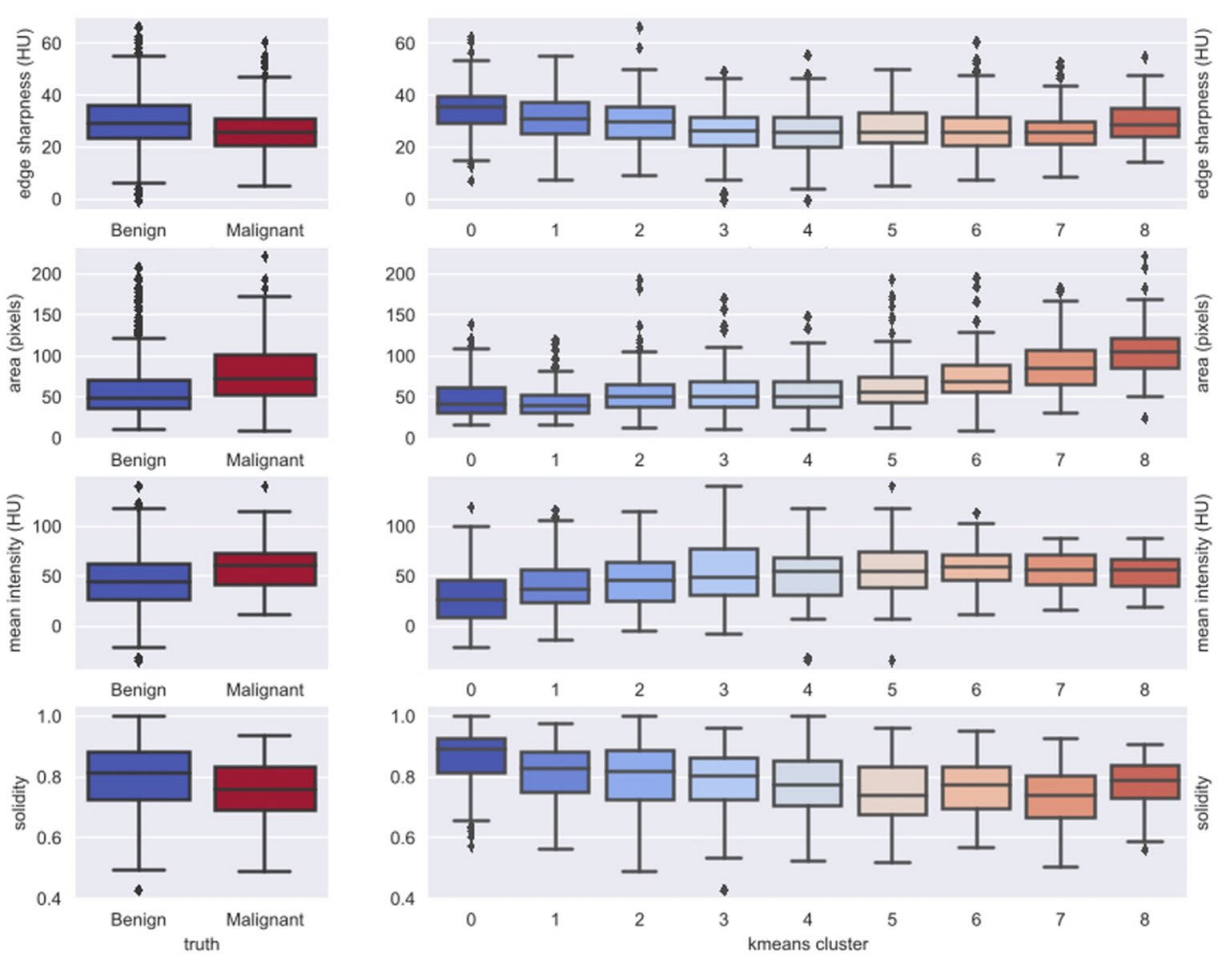
Box plot distributions of known physical features of SHHN against both the ground truth (left sided plots) and against clusters of nodules with similar CNN derived malignancy probability (right sides k-means cluster plots). The differences between benign and malignant SHHN in the left-sided plots were all significant (*p* < 0.0001). In the right sided plots, nodules with higher malignancy probability show a progressive trend to increasing nodule area, decreasing nodule mean attenuation, and less edge sharpness and solidity.

**Figure 4:**
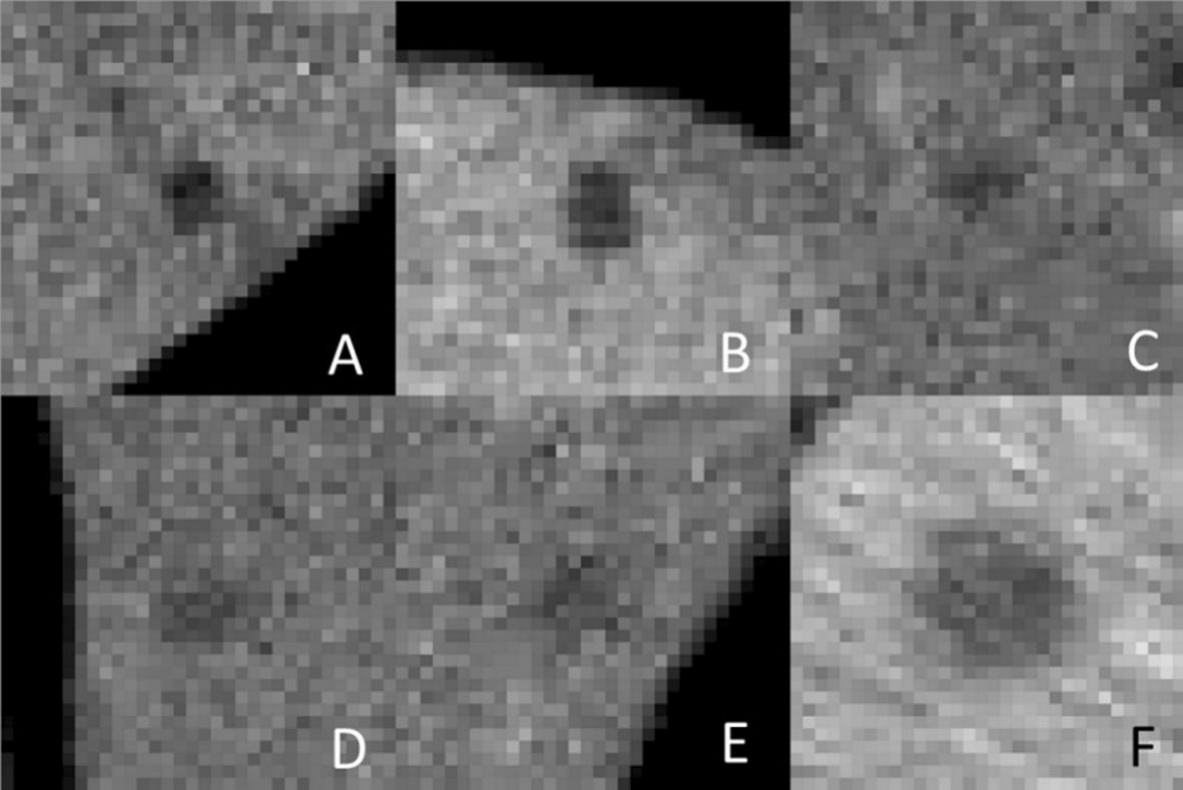
6 selected patches depicting the range of probabilities of malignancy assigned by CNN, (**A**) 1.1%, (**B**) 21.7% (**C**) 40.8% (**D**) 62.6% (**E**) 81.1% (**F**) 96.3%. (**A** & **B** were benign).

Figure 2 and Supplementary figure S3 show the result of embedding the 64 dimensional feature representation of training and testing patches (pre-softmax layer) respectively into the 2-dimensional t-SNE space. Comparison of the nodules in the two extreme ends of the probability scale suggests that malignant nodules are generally larger, of higher attenuation (density closer to enhanced liver and hence less well seen) and less well-defined margins. Figure 3 (left sided box-plots) depicts the boxplot distribution of known physical features of SHHN against both the ground truth (reference standard). There was a significant difference between the mean values of benign and malignant nodule for mean intensity (attenuation, *p* < 0.0001), area (*p* < 0.0001), edge sharpness (*p* < 0.0001) and solidity (*p* < 0.0001). Figure 3 (right sided box plots) also shows the distribution of known physical features in clusters of nodules with similar CNN derived malignancy probability. There is progressive trend to increasing nodule area, decreasing nodule mean attenuation, and less edge sharpness and solidity (Fig. 4), as predicted by prior studies which had used visual inspection.

## Discussion

This study addresses a specific and common clinical problem: that of SHHN in CT scans of patients with known CRC. The phrase “too small to characterize” is one that is commonly seen on CT scan reports and the discovery of SHHN poses a dilemma for patients and physicians^16,17^. We show that despite high diagnostic accuracy of expert radiologists when forced to make a determination, their confidence in characterizing a small hepatic nodule as benign versus malignant was low. In reality, as opposed to this study’s methodology, general radiologists are not forced to make a designation of benign or malignant and therefore many nodules are reported with an equivocal designation, such as “too small to characterize”. Our study therefore highlights the potential value of additional readily and rapidly accessible diagnostic tools that may enhance the diagnostic confidence on CT reporting of small hepatic nodules.

This study used a focused application of CNN to SHHN in patients with a known malignancy. In this study, CNN performed well when assessing SHHN without any clinical information. This was especially promising since the training data was limited; CNN typically requires large training data sets in the order of many thousands of images^8^. Nevertheless, the provision of one additional data point which is detected by the radiologists, the presence of ≥ 1 cm definitive metastases in the liver, improved CNN’s diagnostic performance to equal the radiologists. It is important to note that the CNN diagnostic probability output is not equivalent to confidence rating generated by radiologists in a study setting. However, we believe that the two can be compared to provide insight to the level of certainty for both. CNN rated significantly fewer nodules in the low probability/confidence zone compared to two of the radiologists (Fig. 1). This suggests that CNN may indeed help improve radiologist confidence, thereby expediting patient management. We foresee the use of CNN as an adjunct tool in clinical practice. Upon detecting a SHHN, the radiologists would draw a region of interest about the nodule and indicate whether or not there is presence of a larger definitive metastasis. The CNN then would calculate instantly the probability of malignancy, which can then be incorporated into the report. Our tool can also be integrated into an automated detection algorithm providing both detection and characterization. Importantly, it is likely that our methods with minor refinement are applicable to other malignancies with a propensity for hypovascular hepatic metastases, such as other gastrointestinal, lung and breast adenocarcinomas^3^.

The liver is a complex organ with high degree of normal anatomical variance, a dual vascular supply (hepatic artery and portal vein), profound alterations in structure with chronic disease, and a vast number of primary and secondary neoplasms. It is therefore not surprising that unlike other similar large organs (such as lungs or the brain) there is a relative paucity of studied using automated diagnostic techniques such as radiomics and deep learning, and all utilizing a manually drawn ROI about the lesion. Zhong et al.^18^ and Mokrane et al.^19^ applied texture analysis to their studies to cirrhotic patients using MRI and CT respectively. Both showed improved performance of texture analysis compared to radiologists in determination of benign and malignant cirrhotic nodules. Nodules were ≥ 1 cm in both studies. Yasaka et al. applied CNN to categorize liver nodules on CT into 5 categories, demonstrating a median AUC ROC of 0.92 in differentiating benign from malignant nodules with a mean size of 26.9 mm^15^. They did not limit the patient population to a specific risk but did not compare their model’s performance to radiologists. Using a machine learning-based system, Dankerl et al. assessed automated diagnoses of variety of nodules on CT, also not limited to a specific patient population^13^. Their study showed an accuracy of 81.9% for nodules < 1 cm with in designation of benign vs malignant (compared to 78.3% in this study). The performance of their model improved to 0.92 with input of complementary “high level semantic features” normally found in radiology reports, such as focality, rim continuity, lesion surrounding, and margin definition. The accuracy of our model using a single additional data-point, the presence of additional larger metastases, reached a comparable 90.6%. However, Dankerl et al. did not use an independent testing set, using a leave-one-out analysis which often leads to exaggerated performance due to predisposition to model overfitting^9^.

One criticism of deep learning techniques such as CNN is that its decision-making process is obscured, as a “black-box” process^20^ making detection and correction of errors difficult. To scrutinize its decision-making process, we used t-SNE diagram, the visual scrutiny of which suggested a rational assignment of probabilities (Fig. 2 and Supplementary Figure S3)^9^. Furthermore, we quantified previously known features of benignity and showed significant differences were seen between benign to malignant nodules (Fig. 3)^9^. This correlation between independently derived features of malignancy and their gradation by CNN does not mean that “overfitting” has been eliminated nor that CNN has used the same features but does provide some reassurance that the CNN model behaves logically and applicable to independent real-world data. These suggest that the strong performance of CNN in this study was not due to “overfitting", a machine learning pitfall where “idiosyncratic statistical variations of the training set rather than generalizable patterns”^9^ predominate the results.

This study had some limitations. Our data was derived from one institution with standardized imaging protocols and a single manufacturer of our CT scanners. We would expect increased variability through inclusion of CT scans from other institutions as technical parameters of image acquisition, such as image noise, slice thickness, and intravenous contrast dose and timing, would be different. Our CNN model would possibly have lower performance characteristics and may need to be trained and validated with external datasets. Our training and testing datasets were derived from a select group of patients as is evident from the high prevalence of malignancy in our training and testing set (34.2% and 39.9% respectively) as opposed to lower reported rates of 9.2–14.0% in staging CT scans^3–6^. Consequently, we could not derive positive and negative predictive values in a normal prevalence population. Our patient population was representative of a tertiary referral center with likely higher proportions of aggressive tumors with increased likelihood of metastases. The size of the dataset used for training, in comparison to large data used for CNN, as well as the use of a “leave-one-out approach” increases the predisposition to overfitting and may explain partially why the CNN performance dropped from training set to the testing set. By inclusion of restaging and surveillance scans, we increased the proportion of malignant nodules. Our CNN performance would have to be confirmed or adjusted in a consecutive, non-metastatic-enriched population. Finally, our dataset was relatively small. While the imaging appearance of the 550 SHHN used in this study are likely highly representative, the adjusted probabilities of CNN with hepatic metastatic status were calculated from the 49 patients (138 SHHN) in the testing set. The latter needs to be confirmed in a larger dataset.

In conclusion, we have shown that CNN can equal expert radiologist performance in characterizing SHHN as benign or malignant when adjusting for presence of synchronous ≥ 1 cm hepatic metastasis. We have also shown that CNN resulted in a significantly lower number of nodules rated with low degree of confidence. The use of CNN as an adjunct tool in improving radiologist confidence needs to be investigated.

## Methods

This was a retrospective single center study approved by the institutional Research Ethics Board, waiving the need for patient consent. All methods were performed in accordance to the Government of Canada’s Tri-Counci Policy on Ethical Conduct for Research Involving Humans.

### Patient selection

**Figure 5:**
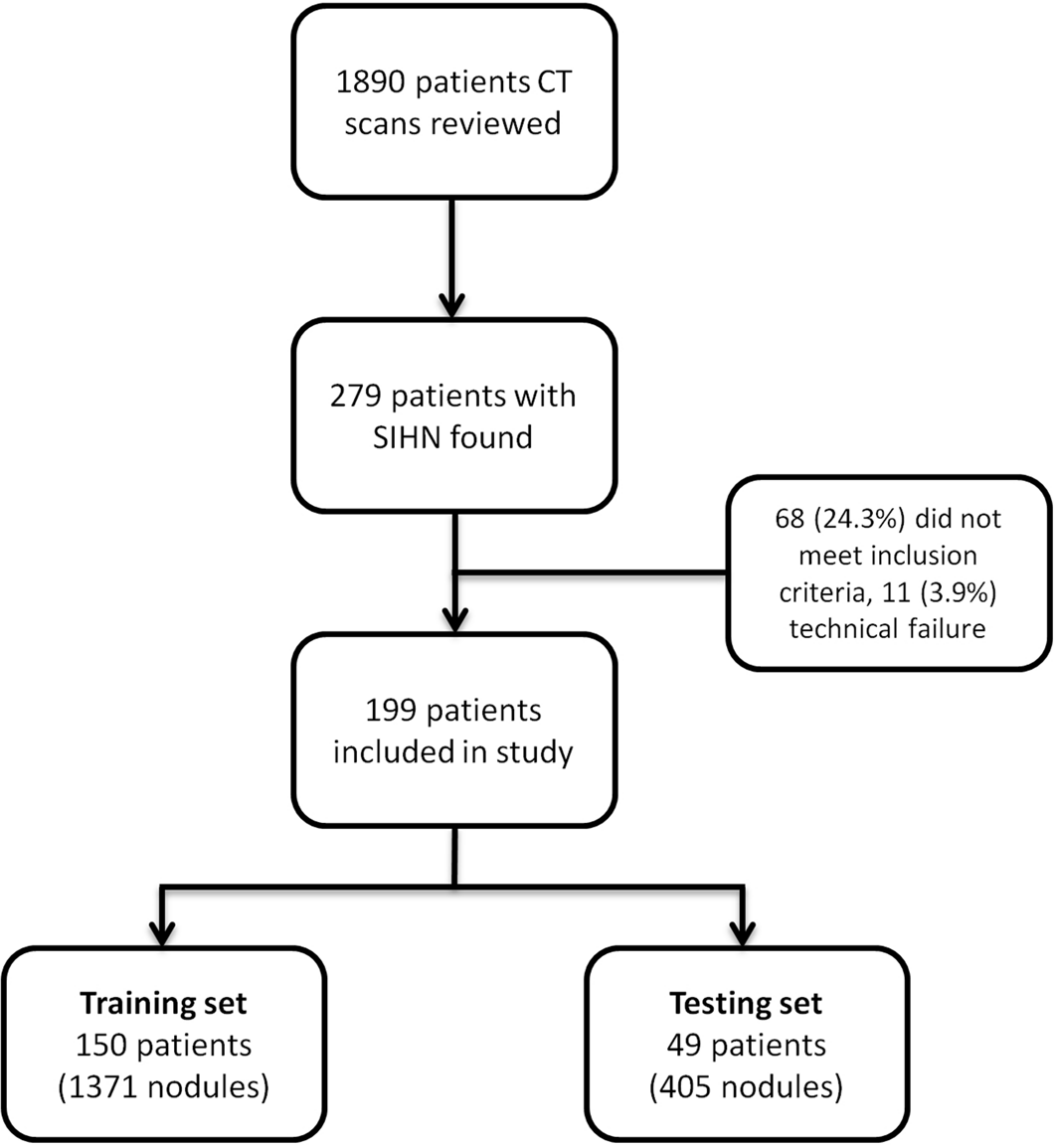
Patient flow diagram.

The records of consecutive patients having CT scans for staging, restaging or surveillance between January 1, 2007 and December 31, 2012 were reviewed for the following inclusion criteria: (1) Histologically proven CRC (2) baseline pre-treatment and follow-up contrast-enhanced CTs all at one institution (3) at least one < 10 mm hypoattenuating liver nodule *reported as indeterminate* on either the baseline scan or subsequent scans (4) the absence of confounding treatment or disease processes (i.e. no systemic or local hepatic treatment prior to scan showing SHHN, second primary malignancy) (5) proof of diagnosis for SHHN. Patients with synchronous metastases were included since hepatectomy would not be necessarily ruled out. Figure 5 depicts patient selection flowchart.

### Patient demographics

One hundred and ninety nine patients with 550 nodules met the inclusion criteria and were included in the study. There were 154 patients with benign nodules only, 38 with malignant nodules only, and 7 with benign and malignant nodules. Benignity was proven by 1 year stability in 159 and by MRI (as hemangiomas) in 2 patients. Malignancy was proven by growth criteria in 30 patients, histopathology in 4, and response criteria in 11. Patients and nodule demographics are summarized in Table 3.

**Table 3:** Patient demographics.

	Training set	Testing set
Patients	150	49
Male: female ratio	1.18	2.40
Mean age	62	60
Age range	20–88	31–84
Nodules	412	138
Mean nodules/patient	2.75	2.81
No. of malignant nodule	141	55
% Malignant nodules	34.2	39.9
Images	1,371	405
Mean number of images/nodule	3.32	2.93
Synchronous metastases ≥ 1 cm in liver	N/A	12/49 (24.4%)
Synchronous metastases outside liver	N/A	6/49 (12.2%)

### Nodule selection

The nodules were reviewed by a senior radiology resident (R.L) , or an abdominal radiologist (M.P, 10-year experience) to ensure they measured < 10 mm, were hypoattenuating and lacked water attenuation (i.e. definite cyst). If multiple nodules were present, no more than 6 were randomly selected to prevent skewing the results.

## Reference standard for proof of diagnosis

Given the heterogeneous management of patients and nodules, a combination of diagnostic standards was used as per past studies^3,4,16,17^. A nodule was considered malignant if there was histopathologic diagnosis, it grew to > 1 cm on follow up contrast-enhanced CT, or responded by ≥ 50% diameter on systemic chemotherapy. A nodule was considered benign if it remained stable for at least 1 year of CT/MRI follow-up or showed definitive benign features on a complementary MRI (for cysts, hemangiomas). Testing set nodules were reviewed by a fellowship-trained abdominal radiologist (KK, 18 years of experience) to confirm appropriate classification.

### Other sites of distant metastatic disease

To provide CNN similar bias as a radiologist, other definitive sites of distant metastatic disease were recorded for the testing set. The CT scans were reviewed (KK) to record synchronous definitive metastatic disease distant to the site of primary CRC. Within the liver, only synchronous metastases ≥ 1 cm were considered as positive. All metastatic disease fulfilled the criteria of histopathologic proof (via biopsy or resection), new development compared to baseline, or growth/shrinkage by 30% in diameter.

### Data preparation and training of the CNN

As CNN typically requires a large set of training data, a decision was made to divide the patient population randomly into a training set inclusive of ¾ of the patients, and testing set of the other ¼. The training of CNN was done by subsequent to and independently of nodule selection and assignment of final diagnosis. A simple schematic of the CNN is shown in Fig. 1. It was made up of 2D convolutional layers and pooling layers in the arrangement depicted. The feature maps were then flattened to form a one-dimensional vector and connected to the fully connected layers. A sigmoid function then outputted a probability of malignancy. The network was trained by feeding in a batch of test images and then comparing the predicted label with the true label. Errors between prediction and ground truth were used to update the weights though back-propagation and the process was then iterated. *Data augmentation* was used to increase the effective size of the training set. We cropped the original image patches of 32 × 32 pixels to produce smaller input images of 24 × 24 pixels which allowed us to translate the image by up to 4 pixels in the horizontal and vertical directions without introducing interpolation artefacts at the edges of the patches.. Together with rotations and reflections, this provided 768 possible variations on a single patch. At each epoch we used oversampling with replacement, followed by random augmentation, to increase the number of benign patches by a factor of 2 and the malignant patches by a factor of approximately 6 in order to provide a balanced training set. Keras 2.0 (https://keras.io/) with a TensorFlow backend (https://github.com/tensorflow/tensorflow) running on a PC equipped with a TitanX GPU (NVIDIA, Santa Clara, Ca) was used.

The hyper-parameters of the CNN were optimized considering the effect on model accuracy of the number of convolutional layers, the number of filters in each layer, the size of the dense layers, number of iterations, and the number of images in each batch fed into the network. To minimize overfitting of the model to the training data, the training data was split into 2 parts; a training set used to train the model and an evaluation set used to assess the model accuracy on an unseen set of images. We therefore stratified the patients in the training set into 5 groups, each group with approximately equal numbers of patients, lesions and patches. All lesions arising from one patient were kept in same group. For each set of model hyper-parameters, we used 4 groups to train our model and left out one group to evaluate the model, and then we repeated this procedure 5 times with a different group used for evaluation each time. As our dataset is small, we chose a small network size and explored between 2–4 convolutional layers, with between 16–32 filters in each layer. The final dense layer was varied between 32–128 units. Once the best hyper-parameters were identified, 5 models, each one trained on 4 out of the 5 groups of training data, were saved.

From the training stage we found that the following configuration shown in Supplementary materials A (supplementary Fig. 1) gave the highest accuracy: The CNN is composed of 2 convolutional layers, each with 16 filters, followed by a max pool layer, followed by another 2 convolutional layers each with 32 filters and a second max pooling layer. A single dense layer of 64 nodes is surrounded by 50% dropout layers to improve generalizability. The model definition is provided in Supplementary Materials B. We tested batch sizes of 40, 60 and 80 and varied the number of epochs between 20 and 600; a batch size of 80 images and training for 50 epochs were found to give the best results. This resulted in an average AUC of 0.854 ± 0.045 when the patches were evaluated individually and 0.892 ± 0.038 when the patches for each lesion were aggregated.

Threshold of 0.5 was used to differentiate benign from malignant nodules as it provided the best combination of sensitivity and specificity (see Supplementary Table 1 in Supplementary Materials A).

### Testing CNN vs radiologists

Three fellowship-trained abdominal radiologists independently reviewed the anonymized CT. The radiologists (T.K.K, H.J, and S.A.J) had 19, 17, and 7 years of experience working in oncological/hepatobiliary center. They were aware of history of CRC but blinded to the diagnosis of individual liver nodules. The complete scans with nodule coordinates (image number and liver segment) were provided. The readers were asked to first assign the diagnosis (benign or malignant) and then estimate their confidence on a 5-point Likert scale. The radiologists were instructed explicitly to consider the nodules as they normally would, that is to search for contextual factors including synchronous metastases and make their decision as per their routine clinical practice.

### Comparison of diagnostic probability/confidence between CNN and radiologists

The radiologists’ diagnosis and confidence ratings were converted to a 10 point ordinal scale (i.e. benign with (high) confidence of 5 was designated as 1, benign with (low) confidence of 1 was designated as 5, malignant with (low) confidence of 1 was designated as 6, malignant with (high) confidence of 5 was designated as 10)0.1. Similarly, CNN’s derived malignant probability fraction (between 0 and 1) were converted into an ordinal scale (0–0.10%, 1; 0.11–0.20%, 2; etc.). For both radiologists and CNN, a low probability/confidence rating was defined as nodules with ratings of 4–5 (for benign nodules) and 6–7 (for malignant nodules).

### Visualizing the CNN

To assess correlation of CNN categorization to real world data, the training data were analyzed for previously described differentiating imaging features of benign and malignant nodules using K-means clustering technique. These features were nodule size (smaller size in benign), attenuation (density, lower in benign), and discrete margins (more discrete in benign) (3). Size was determined by measuring nodule area and attenuation through mean attenuation values of the central 9 pixels. Margins were assessed by two means, edge-sharpness (mean attenuation difference between outside and inside of the nodule along the margins), and solidity (a measure of irregularity along the margin of the nodule). (see Supplementary Materials C and supplementary Fig. 2). Upon the completion of the study, the pre-softmax layer of the training and testing sets were depicted on t-Distributed Stochastic Neighbor Embedding (t-SNE) plots. T-SNE plots are means of depicting the relationship of multidimensional objects into a two-dimensional representation^9^.

### CT scans

Acquisition parameters of CT scans are noted in Supplementary Materials D.

### Statistical analysis

Binary logistic regression was performed to identify the combination of the performance of CNN-derived probability and presence of metastasis in liver in predicting the malignancy of liver nodules. A similar analysis was performed with different radiologists’ confidence rating predicting the malignancy of liver nodules. ROC curve analysis was performed using a nonparametric approach^21^. Sensitivities and specificities were reported at a threshold point of 0.5 for both radiologists and CNN. Agreement between radiologists and CNN was assessed using unweighted Kappa values by converting CNN probabilities into a binary scale (benign/malignant). Agreement was interpreted using the following scale: 0.41–0.60 as moderate, 0.61–0.80 as substantial, and > 0.81 as almost perfect. McNemar’s Test was used to compare proportion of nodules rated with low confidence by radiologists to CNN. All tests were two-sided. A *p* value < 0.05 was considered statistically significant. Analysis was performed using SAS (v.9.4 windows, SAS Institute, Cary, North Carolina).

## Supplementary information

Supplementary Information.

## Abbreviations

AUC ROC: Area under curve of receiver operating curve
AI: Artificial intelligence
CRC: Colorectal carcinoma
CT: Computed tomography
CI: Confidence interval
CNN: Convolutional neural networks
SHHN: Small hypoattenuating hepatic nodules
