# Supplementary material for: Convolutional neural networks versus radiologists in characterization of small hypoattenuating hepatic nodules on CT: a critical diagnostic challenge in staging of colorectal carcinoma
Source: Sci Rep. 2020 Sep 17;10:15248. doi: 10.1038/s41598-020-71364-5 (PMC7499427; doi:10.1038/s41598-020-71364-5)
Supplement: Supplementary file 1 — Supplementary Information. [file 41598_2020_71364_MOESM1_ESM.pdf]

SUPPLEMENTARY MATERIALS:

Convolutional neural networks versus radiologists in characterization of small hypoattenuating hepatic nodules on CT: A critical diagnostic challenge in staging of colorectal carcinoma

Korosh Khalili[1,2] MD Korosh.khalili@uhn.ca,

Raymond L. Lawlor[1,2] MD Raymond.lawlor@mail.utoronto.ca

Marina Pourafkari[1,2] MD Marina.pourafkari@uhn.ca

Hua lu[1,3,] , BSc henryhua.lu@mail.utoronto.ca

Pascal Tyrrell[1,3,] PhD pascal.tyrrell@utoronto.ca

Tae Kyoung Kim[1,2] MD TaeKyoung.Kim@uhn.ca

Hyun-Jung Jang[1,2] MD Hyun-Jung.Jang@uhn.ca

Sarah A. Johnson[1,2] MD Sarah.johnson@uhn.ca

Anne L. Martel[4] PhD a.martel@utoronto.ca

[1]Department of Medical Imaging, University of Toronto, Toronto, ON, Canada

[2] Joint Department of Medical Imaging, University Health Network, Sinai Health System, Women's College Hospital, Toronto, ON, Canada

[3]Department of Statistical Sciences, University of Toronto, Toronto, ON, Canada

[4]Department of Medical Biophysics, University of Toronto, Toronto, ON, Canada

Corresponding Author:

Korosh Khalili, MD, FRCPC, MHSc, Joint Department of Medical Imaging

University Health Network, Princess Margaret Cancer Centre

610 University Ave, Toronto, ON. Canada, M5G 2M9

Korosh.khalili@uhn.ca   Phone (1)416 9464501 ext. 4833, Fax (1) 416 9466564

SUPPLEMENTARY MATERIALS A:

CNN architecture and sample performance with various thresholds

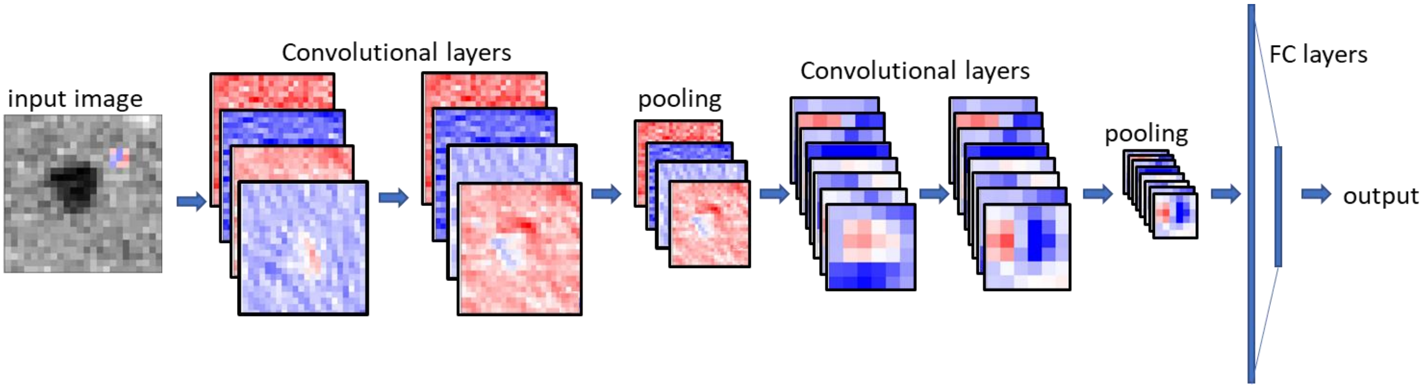

Supplementary figure S1. CNN was made up of 2 convolutional layers, each with 16 filters, followed by a max pooling layer and then another 2 convolutional layers each with 32 filters and a second max pooling layer. The final dense layer had 64 elements.

Supplementary Table S1

Diagnostic performance of CNN using the training set data based on various threshold values used to separate mean probability of benign vs malignant nodules.

Threshold (0-1)	Sensitivity	Specificity	Positive Predictive value	Negative predictive value
0.5	85.7	83.1	63.4	94.4
0.3	92.4	70.7	51.9	96.4
0.1	96.2	40.4	35.6	96.9

SUPPLEMENTARY MATERIALS B:

CNN model definition code.

```
from keras.models import Sequential

from keras.layers import Conv2D, MaxPooling2D

from keras.layers import Activation, Dropout, Flatten, Dense


def create_model(train_shape):

    model = Sequential()

    model.add(Conv2D(16, (3, 3), input_shape=train_shape))

    model.add(Activation('relu'))

    model.add(Conv2D(16, (3, 3), input_shape=train_shape))

    model.add(Activation('relu'))

    model.add(MaxPooling2D(pool_size=(2, 2)))

    model.add(Conv2D(32, (3, 3)))

    model.add(Activation('relu'))

    model.add(Conv2D(32, (3, 3)))

    model.add(Activation('relu'))

    model.add(MaxPooling2D(pool_size=(2, 2)))


    model.add(Dropout(0.5))


    model.add(Flatten())

    model.add(Dense(64))
```

```
model.add(Activation('relu'))
model.add(Dropout(0.5))
model.add(Dense(1))
model.add(Activation('sigmoid'))

model.compile(loss='binary_crossentropy',
        optimizer='adam',
        metrics=['accuracy'])
return model
```

SUPPLEMENTARY MATERIALS C:

Extracting described differentiating features from SHHN patches for K-means clustering

The training data, yielding 1371 patches, were used for extraction of the previously described differentiating features between benign and malignant SHHN. Manipulation of the images and patch extraction were carried out using Python (version 3.6, Python Software Foundation. Available at http://www.python.org) and the SimpleITK module (available at www.simpleitk.org). In order to calculate features such as lesion size, mean intensity, edge sharpness and solidity, segmentation was first performed. The radiologist manually marked a bounding box centered on the lesion of interest and a margin of normal tissue pixels was included around the edge of the lesion. To carry out automatic segmentation we first determined the mean intensity of the 3x3 patch at the center of the bounding box (*mc*) and the mean intensity of the pixels at the edge of the bounding box (*me*) box. A threshold was set at an intensity of (*mc* + *me*)/2 and then any pixels, connected to the central region, with intensities below this threshold were labeled as lesion. We used morphological filtering to remove any holes within the region of interest. This approach failed in approximately 25% of lesions as many of the lesions were close to the edge of the liver; we used a method developed for segmentation of pulmonary lesions to handle cases where the region of interest "leaked" into the tissue surrounding the liver [20]. Finally, a visual inspection was carried out and 184/1371

(13.4%) of the patches were filtered out due to poor segmentation quality, mostly due to very poor signal to noise ratio in the image.

T-SNE plots and K-means clustering were performed using Python. The number of clusters was determined using the t-SNE plot. Nine was the minimum number of clusters that provided a clear separation between the two clusters at the top of the plot (see Supplementary figure 2).

Supplementary figure S2. t-SNE plot used to determine number of k-means clusters

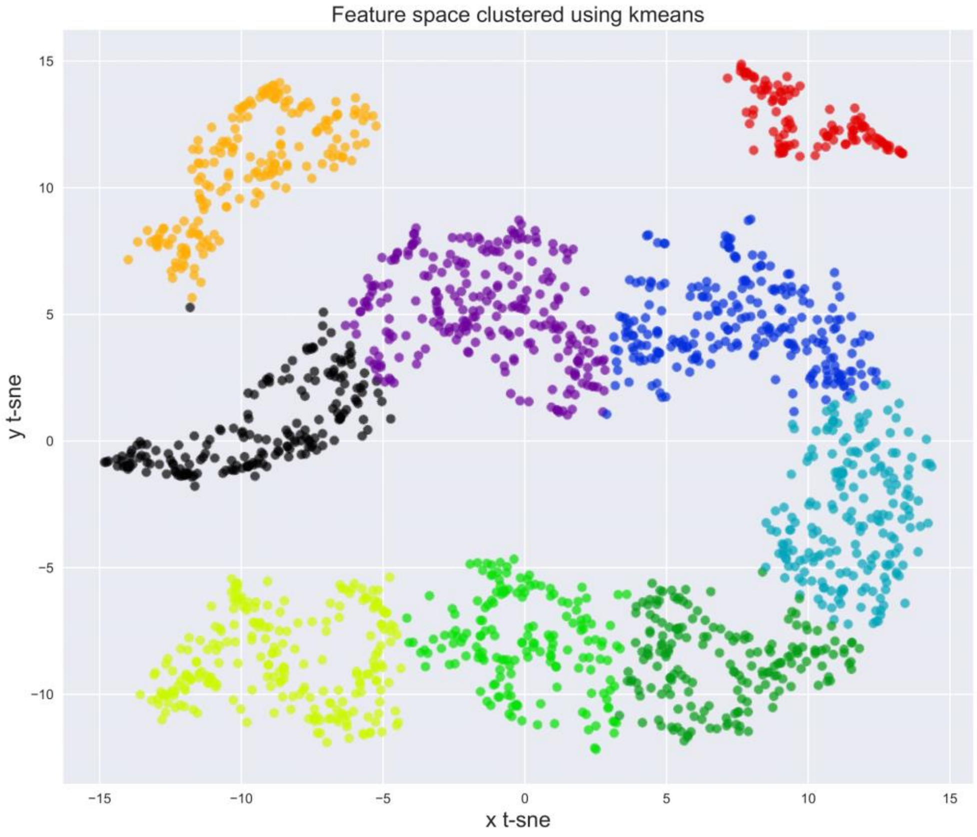

SUPPLEMENTARY MATERIALS D

CT Technique

All CT scans were performed on 64 or 320 multidetector scanners (Aquilion 64 or Aquilion One, Canon (formerly Toshiba) Medical Systems Corporation, Otawara, Japan). Images were acquired using 120 kVp, gantry rotation speed of 0.35-0.75s and automatic mAs setting with standard deviation of 13-15 Hounsfield units. The volumetric acquisition was reconstructed at 5 mm slice thickness every 2.5mm in the axial plane and 3 mm thickness every 3 mm in the coronal and sagittal planes (the sagittal performed after January 2010). The manufacturer's standard soft tissue filter (FC04) was used for the reconstruction. Intravenous contrast consisted of 100 cc of iodixanol (320mg/ml, GE healthcare, Chicago, IL) injected at 3 cc/s. The abdomen was then imaged 70 seconds after initiation of contrast bolus injection.

Supplementary Table S2 Per-nodule results of radiologists, CNN and CNN with liver metastatic status in prediction of malignant SHHN.

	True Positive	True Negative	False Positive	False Negative
Rad 1	49	74	10	5
Rad 2	46	76	8	8
Rad 3	53	64	20	1
CNN	44	65	19	10
CNN & Liver Metastatic Status	44	81	3	10

Supplementary Table S3

Results of agreement analysis between various readers.

Reader	Kappa (benign/malignant)
Radiologist 1 vs CNN	0.70
Radiologist 2 vs CNN	0.67
Radiologist 3 vs CNN	0.57
Radiologist 1 vs 2	0.78
Radiologist 1 vs 3	0.60
Radiologist 2 vs 3	0.52

Supplementary figure S3, (A)   t-SNE plot depicting probability of malignancy from training data with blue representing a benign prediction and red representing a malignant prediction. Note the areas of highest assigned probability of benignity (darkest blue) and malignancy (darkest red). (B) Plot of image patches of training. The color of boxes outlining the patch indicate actual diagnosis (blue: benign; red: malignant).

SUPPLEMENTARY FIGURE S3A

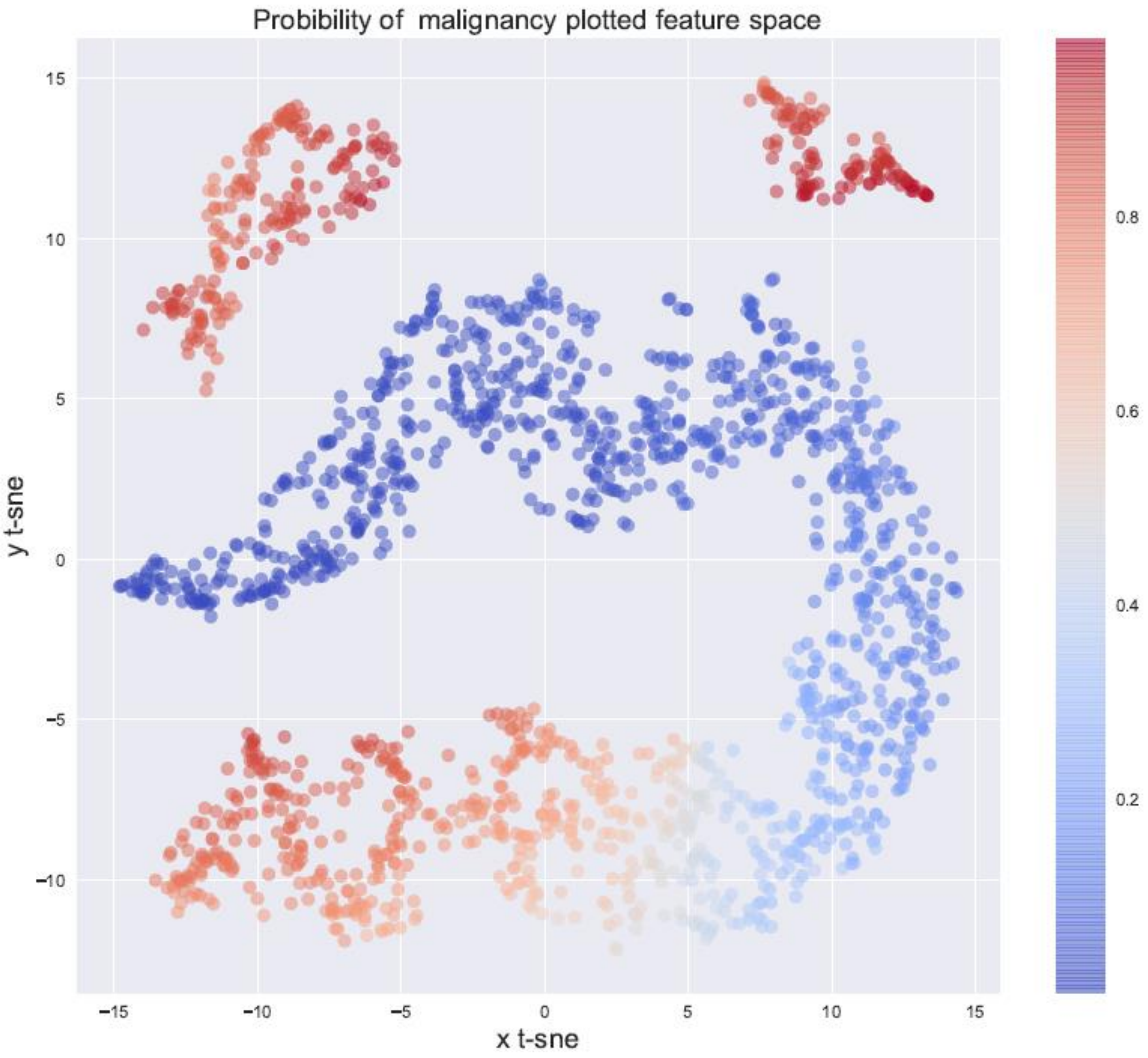

SUPPLEMENTARY FIGURE S3B

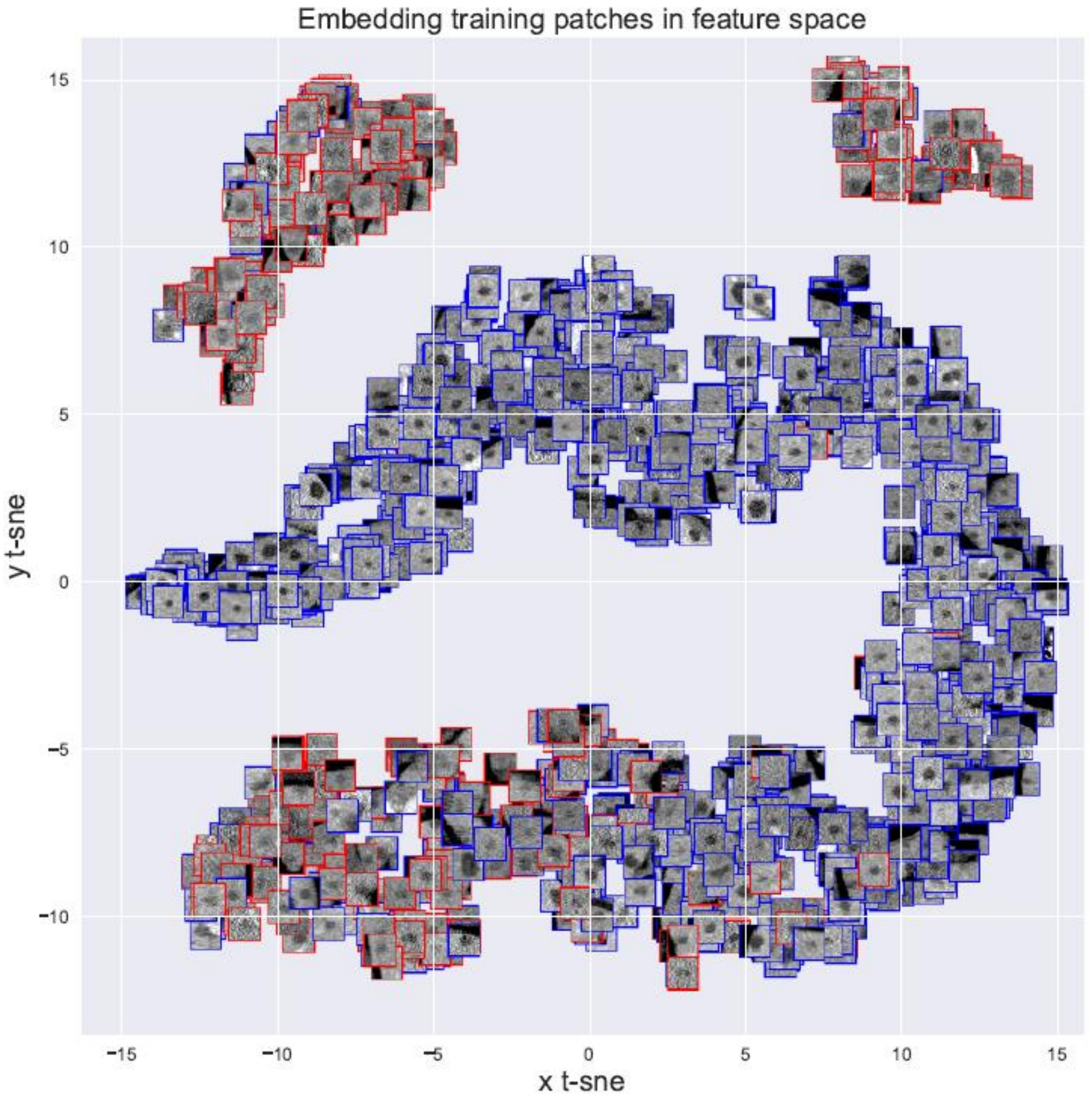

:
